# Scale Up Pillaring: A Study of the Parameters That Influence the Process

**DOI:** 10.3390/ma10070712

**Published:** 2017-06-27

**Authors:** Francine Bertella, Sibele B. C. Pergher

**Affiliations:** Laboratory of Molecular Sieves—LABPEMOL, Institute of Chemistry, Federal University of Rio Grande do Norte—UFRN, Av. Senador Salgado Filho, 3000, Lagoa Nova, University Campus, 59072-970 Natal-RN, Brazil; francinebertella@gmail.com

**Keywords:** clays, Al-PILC, pillared clays, scale up

## Abstract

Pillared clays (PILCs) are interesting materials mostly due to their high basal spacing and surface area, which make them suitable for adsorption and catalysis applications, for example. However, the production of these materials on industrial scale is dependent on research about what parameters influence the process. Thus, the objective of this work was to evaluate what parameters influence the pillaring procedure. For this, pillared clays were synthesized following three series of experiments. In the first series, the effect of the amount of water in a clay suspension was evaluated. The best results were obtained by using diluted suspensions (1 g of clay to 100 mL of water). In the second series, several pillaring methods were tested. In the third series, the amount of pillared clay was raised to 50 g. Fifty grams of pillared clay can be obtained using the pillaring agent synthesized at 60 °C with further aging for 24 h, and this material exhibited high basal spacing (17.6 Å) and surface area (233 m^2^/g). These values are comparable with the traditional pillaring method using only 3 g of clay.

## 1. Introduction

Clays are abundant natural materials with a low cost. To improve the catalytic and adsorptive properties of these materials, clays can be submitted to different processes, such as pillaring and acid activation. Usually, clay pillaring occurs through cation exchange of natural cations (Ca^2+^, Na^+^, Mg^2+^) present between clay layers for bigger cations such as polyhydroxy cations of Al (a pillaring agent). These larger cations act as pillars, separating the clay layers, increasing the basal space and creating microporosity. The calcination provides stability to the pillared clay, and the resultant material presents small cavities and a large surface area. These properties, combined with the low cost of clay, make pillared clays alternative catalysts to zeolites.

The majority of articles found in the literature use polyhydroxy cations (mostly aluminum, zirconium, iron, and titanium) as pillaring agents [1,2,3]. These cations can be used separately (just one type of pillar) [4,5,6,7,8,9,10] or as mixed pillars [11,12,13,14,15,16,17].

However, most papers about pillared clays report the use of polyhydroxy cations of only aluminum as the pillaring agent. Solutions containing this complex are made through addition of a base (hydroxide or carbonate) to a salt of aluminum (AlCl_3_ or Al(NO_3_)_3_) until a molar ratio of OH/Al = 2.5 is attained or through dissolution of powdered Al in AlCl_3_ [1]. Solutions prepared with these methods contain mostly three species: hydrated monomeric aluminum, the polyhydroxy cation [Al_13_O_4_(OH)_24_(H_2_O)_12_]^7+^, also known as ε-Keggin ion or Al_13_, and polynuclear aluminum [18,19,20]. Some of these Al-pillared clays have been reported as support for active phases, such as metals, in catalytic reactions [21,22,23].

Variations in the synthesis provide different characteristics to the resulting materials, and the most important parameters that can be varied are related to the formation, intercalation, and posterior fixation of the polynuclear cations between the clay layers. No general rules exist about the best conditions for the synthesis [24].

Several pillaring methods and pillaring solutions have been reported in the literature. Despite the major studies that have been performed on a laboratory scale (with small quantities of clay), some authors have utilized concentrated suspensions to increase the scale of pillaring [14,25,26,27,28,29,30,31]. In this sense, to reduce the volume of water and intercalation times, Sanabria et al. [14] have performed pillaring procedures by using dialysis membranes and ultrasound to produce pillared clays with mixed Al-Fe, Al-Ce-Fe pillars. By this methodology, they were able to produce pillared clays with similar features to those of the solid synthesized by their conventional procedure.

Nevertheless, a closer look into the literature reveals that an enormous amount of parameters can influence the pillaring processes, like the starting clay material, the synthesis condition and nature of the pillaring agent, the use of concentrated or dilute clay suspensions, time and temperature of intercalation and conditions of washing, drying and calcining processes. In this view, to study a scale-up pillaring process it is of primary importance the optimization of key parameters in order to obtain pillared clays with comparable features (basal spacing and surface areas) similar to the material synthesized by the conventional procedure. To achieve this purpose, a systematic study of the most important variables that influence a scale-up pillaring process, like pillaring method, concentration of clay suspension and amount of clay, using the same starting clay is of paramount importance.

Thus, the aim of the present study is to prepare and characterize pillared clays modifying the conditions of the synthesis to determine which parameters influence the increase in the pillaring scale of a bentonite clay.

## 2. Materials and Methods

### 2.1. Traditional Pillaring Method

A bentonite clay consisting of mostly montmorillonite, supplied by Colorminas Colorifício e Mineração S/A, Brazil, was used in this study. The chemical composition of the pristine clay is: 83.02% Si, 13.55% Al, 1.01% Ca, 1.67% Mg, 0.25% Na and 0.50% K. The cation exchange capacity (CEC) determined for the natural clay is 155 mEq/100 g.

The first pillaring stage is the preparation of the pillaring solution. In this way, 500 mL of a NaOH solution (Sigma-Aldrich, St. Louis, MO, USA) and 250 mL of a AlCl_3_·6H_2_O solution (Vetec Fine Chemicals, Duque de Caxias, Brazil), both 0.2 mol/L, were used in order to prepare the pillaring agent. The NaOH solution was dripped slowly into the Al solution (approx. 1 mL/min) under constant stirring at ambient temperature. Then, the pillaring solution was maintained under these synthesis conditions for 6 days. The OH/Al ratio was 2, and 15 mEq Al/g of clay was used [2].

After the 6 days of aging the pillaring solution, 3 g of clay was stirred into 300 mL of distilled water (1 g/100 mL) for 2 h at room temperature to hydrate the interlayer cations and expand the lamellae.

Subsequently, the pillaring agent was added to the clay suspension, and the mixture was stirred for 2 h at room temperature to allow the natural clay cations to be exchanged with the prepared polyhydroxy cations (pillaring agent). The material was vacuum-filtered (using a Büchner funnel and filter paper serving as the porous barrier), washed abundantly with distilled water, dried overnight in an oven at 60 °C and finally calcined at 450 °C for 3 h in a muffle furnace (the heating rate was 5 °C/min).

This pillaring procedure has been performed several times to prove its reproducibility.

### 2.2. Scale Up Pillarizations

The experiments were divided into three series. In series 1, the effect of water during the expansion of clay was studied. In series 2, the method of pillaring was studied, and in series 3, the amount of pillared clay was increased.

#### 2.2.1. Series 1: Effect of Water

To evaluate the effect of water utilized to expand the clay, several methods were tested (Table 1). The OH/Al = 2 ratio and 15 mEq Al/g of clay were maintained in all experiments.

Methods 1, 2, and 3 were performed according to the traditional procedure, only modifying the amount of water present in the clay suspension. However, methods 4 and 5 were performed in a distinct form, with the exception of the synthesis of the pillaring solution. In method 4, after preparation of the clay suspension (10 g of clay with 1 L of distilled water), 500 mL of water was removed by centrifugation (5000 rpm for 10 min). Later, the remaining suspension was added to the pillaring solution, and the mixture was stirred for 2 h. The clay was vacuum-filtered, washed with distilled water, dried in an oven at 60 °C, and calcined at 450 °C for 3 h, as previously described.

Method 5 was performed similarly to method 4, but after the stage of hydration of the interlayer cations, 900 mL of water was removed from the clay suspension by centrifugation. The remaining 100 mL of clay suspension was added to the pillaring agent performing the pillaring stage similarly to the other experiments. This procedure (removal of water suspension) was performed to verify the influence of the amount of water utilized in the clay suspension.

All methods from this series have been performed more than once in order to check their reproducibility.

#### 2.2.2. Series 2: Method Effect

In this series of experiments, several pillaring methods were tested (Table 2). In method 6, 150 mL of a NaOH solution and 75 mL of a AlCl_3_·6H_2_O solution, both 0.6 mol/L, were utilized to prepare the pillaring solution. The NaOH solution was dripped slowly (1 mL/min) into the Al solution under constant stirring at ambient temperature and maintained under these conditions for 6 days. Subsequently, the pillaring agent was transferred to a round bottom flask containing 3 g of dry clay and kept under reflux for 24 h at 80 °C. Then, the material was vacuum-filtered, washed with distilled water, dried in an oven at 60 °C, and calcined at 450 °C as previously described in Section 2.1.

Experiment 7 proceeded as described in the traditional method. However, after 6 days of aging, the pillaring solution was stored for a month in an amber vial to check whether the properties of the pillaring solution remained over the course of time. The pillarization procedure was performed as described in the traditional method.

An in situ pillarization, tested in method 8, was performed differently from all other methods. Ten grams of clay was added to 250 mL of an AlCl_3_·6H_2_O solution (0.6 mol/L) and stirred for 2 h. Subsequently, 500 mL of a NaOH solution (0.6 mol/L) was dripped slowly (1 mL/min) into the Al solution and stirred for 6 days at ambient temperature. After the aging time, the material was washed, filtered, dried, and calcined as previously described in Section 2.1.

Experiment 9 was performed as described previously. However, the clay was first expanded with water (3 g in 300 mL) for 2 h at ambient temperature. After the solution of aluminum chloride (250 mL of 0.44 mol/L) was added to the clay suspension, the mixture was stirred for 2 h. This solution had to be more concentrated to maintain the final concentration at 0.2 mol/L after the solution had been added to the clay suspension. The following procedures were performed as described in the previous method.

Pillaring method 10 was performed as described by Leite et al. [32]. To prepare the pillaring agent, 500 mL of a NaOH solution and 250 mL of an AlCl_3_·6H_2_O solution, both 0.2 mol/L, were utilized. However, during the dripping (1 mL/min), the solution was kept at 60 °C with stirring. Then, the solution was stirred at ambient temperature for 24 h. Subsequently, 3 g of clay was dispersed in 150 mL of water for 48 h of stirring at ambient temperature. Then, the pillaring agent was added to the clay suspension and stirred for 48 h more. Finally, the clay was filtered, washed, dried, and calcined as previously described in Section 2.1.

Method 11 was performed in the same manner as method 10. However, the stirring time was reduced from 48 to 2 h. The same procedure was performed with the cation exchange stage: the pillaring solution and the clay suspension were kept in contact for just 2 h. The pillaring agent synthesis was performed as described in method 10, without modifications.

The majority of methods from this series have been performed just once.

#### 2.2.3. Series 3: Increase in the Amount of Pillared Clay

In this series of experiments, the amount of pillared clay was increased. Table 3 presents the modified parameters for each experiment. In all methods, 1 g of clay to 100 mL of water was utilized.

Pillaring methods 3, 12, 14 and 15 were performed in the traditional manner: the pillaring agent was stirred for 6 days at ambient temperature. The difference between the methods is the concentration of the solution to maintain the relation of 15 mEq Al/g of clay.

In method 14, solutions of 1.5 mol/L were utilized. Due to this high concentration, the pillaring solution became turbid, requiring stirring for 13 days, but even after this period, the pillaring solution still presented some turbidity.

In methods 11, 13 and 16, the pillaring solution was kept at 60 °C during the dripping (1 mL/min) of the sodium hydroxide solution into the aluminum chloride solution. Then, the pillaring agent was stirred at ambient temperature for 24 h. The following procedures were performed as described above.

All methods from this series have been performed more than once in order to check their reproducibility.

#### 2.2.4. Characterization

The prepared materials were characterized by X-ray diffraction (XRD) on a Shimadzu-XRD-7000 apparatus (Shimadzu, Kyoto, Japan) using Cu radiation (λ = 1.54 Å) and 0.02° step size. N_2_ physisorption isotherms were measured using a Quantachrome-NOVA 2200e apparatus (Quantachrome, Boynton Beach, FL, USA). Prior to analysis, the samples were degassed for 3 h at 300 °C under vacuum. The surface areas were obtained using the Brunauer, Emmet and Teller (BET) method, and the micropore volumes and external areas were calculated by the t-plot method using the Harkins-Jura-de Boer t-equation [33]. The total pore volume was calculated at a partial pressure *p*/*p*_0_ of 0.95. The micropore area was calculated as the difference between the BET and external surface areas.

## 3. Results and Discussion

### 3.1. Series 1: Effect of Water

**Figure 1:**
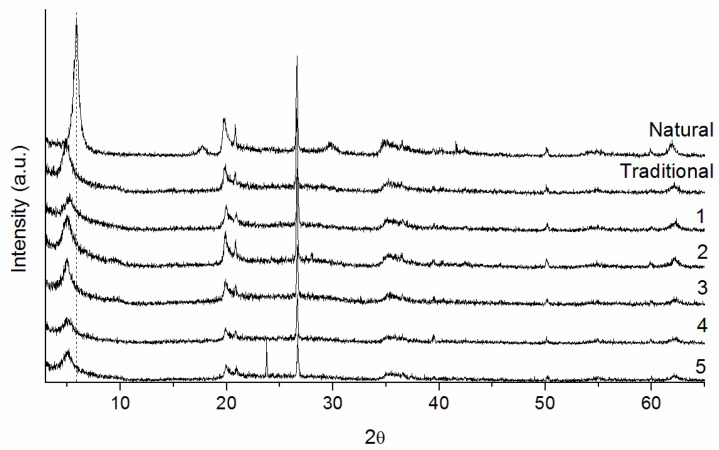
X-ray diffractograms of the natural, traditional and 1 to 5 (series 1) pillared clays.

Figure 1 shows the X-ray diffraction patterns of the natural clay, the pillared clay using the traditional method and the samples of series 1 (1 to 5). Regarding to the natural clay, the material composition is based mostly on montmorillonite (2θ = 5.8°; 17.7°; 19.8° and 35.1°) and quartz (2θ = 26.6°) [34].

The shift of the first reflection, which was due to the (001) plane, to smaller angles (2 theta axis) shows that the basal spacing increased (Table 4), indicating that the clay cations were exchanged for the prepared polyhydroxy cations.

The pillared clays using the traditional methods (2 and 3) presented more intense (001) reflections compared to samples 1, 4, and 5; indicating a more organized structure of the lamellae. This more organized structure is reflected in the values of the basal spacing and the surface areas obtained (Table 4). Sample 1 presented the lowest values of surface area (142 m^2^/g) and basal spacing (16.8 Å) due to the amount of water used to expand the clay (100 mL). In methods 4 and 5, 500 and 900 mL of water, respectively, were removed before the stage of pillarization. As a consequence of this removal of water, these samples presented lower surface areas (149 m^2^/g) in comparison with PILCs obtained by methods 1, 3 and traditional (higher than 200 m^2^/g). Therefore, the amount of water used to expand the lamellae also exerts a great influence in the cationic exchange of natural clay cations by the prepared oligomers, translating into a decrease in surface area when fewer amounts of water (methods 4 and 5) are used. Similar results were obtained with samples 2 and 3, indicating that the minimum relation of clay/water to obtain materials with elevated basal space and surface area (above 200 m^2^/g) is 1/50.

**Figure 2:**
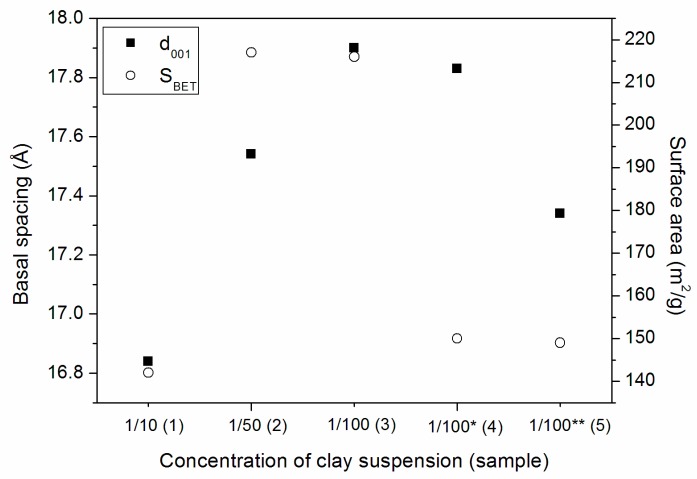
Relation between the concentration of the clay suspension to basal space (Å) and surface area BET (m^2^/g) for series 1 samples. * 500 mL and ** 900 mL of water removed before pilarization.

Figure 2 presents a comparison between the basal spacing, the concentration of the clay suspension and the surface area BET of this series of samples. The best results were obtained through method 3, where elevated amount of water in the clay suspension was used (1/100) and was maintained until the end of the process. For samples 4 and 5, a drastic reduction of surface area occurred due to the removal of water from the clay suspension before the stage of pillaring, which proves that the high quantity of water (diluted suspensions) generates materials with elevated basal spacing and surface areas.

Table 5 presents the textural parameters (BET surface area, micropore area, external area, total pore volume and micropore volume) of series 1 materials.

When the pillared clay prepared by the traditional method is compared with natural clay, the external area values, which include the external surface plus the meso and macropore area, did not vary. The micropore volume is approximately 10 times larger in the pillared clay due to the increase in basal space. Because of this increase in the micropore volume, the micropore area is approximately 10 times larger. This area is responsible for the increase in total area (BET) in the pillared material. Methods 4 and 5 presented the closest results to those when using the traditional method, with surface areas above 200 m^2^/g, a micropore area of 180 m^2^/g and a pore volume (total and micro) similar to the related values in the literature [2,35].

### 3.2. Series 2: Method Effect

**Figure 3:**
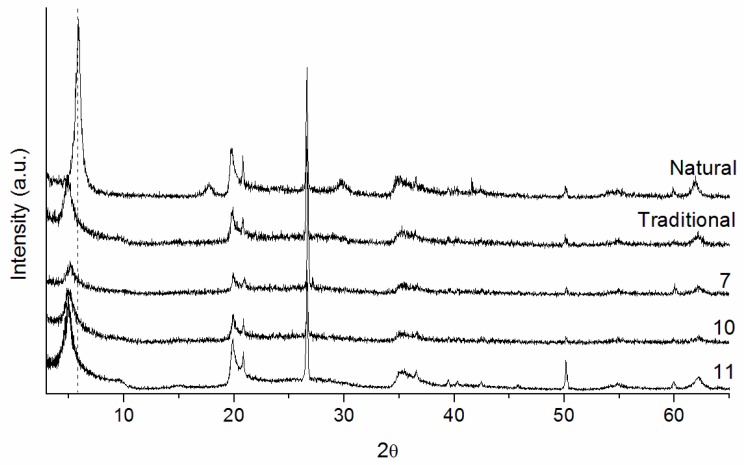
X-ray diffractograms of the natural clay, traditional pillared clay and 7, 10 and 11 pillared clays (series 2).

In this series, several pillaring methods were evaluated according to basal spacings, and surface areas obtained. The pillaring methods that employed reflux (sample 6) and in situ processes (samples 8 and 9) resulted in low basal spacings and low surface areas, indicating that these methods were not effective (Table 6). Samples 7, 10, and 11 presented larger basal spacings than the natural clay (Figure 3).

Method 7, in which the pillaring agent was allowed to stand for a month and used subsequently, resulted in high surface area and basal spacing, indicating that the properties of the pillaring agent had not changed over time, proving that large amounts of pillaring agent could be produced and stored without the pillaring solution losing its function.

Samples 10 and 11, in which the pillaring agent synthesis was performed in one day with heating at 60 °C, presented high basal spacings and surface areas, demonstrating the insertion of Al pillars. Thus, in the methods in which the pillaring agent was prepared separately (without being in situ) and with a clay expansion in water, the best results were obtained. Therefore, the pillaring method influences the characteristics of the materials obtained, and when comparing methods 10 and 11, there is no need to expand the clay lamellae and to submit the clay to cationic exchange for 48 h. These stages are fast, so 2 h is sufficient for each procedure (clay expansion and cationic exchange) to obtain high basal spacings and surface areas.

Table 7 presents the textural parameters obtained using methods 7, 10, and 11, which represent the highest values of basal spacings of the series 2 samples. The samples prepared using methods 7, 10, and 11 are compared to the natural clay and the sample pillared by the traditional method (Table 7).

The samples prepared by methods 10 and 11 presented similar features in comparison with the clay pillared by the traditional method. The surface areas obtained by these materials are in the same order (225 and 237 m^2^/g) than the PILC prepared by the traditional pillaring procedure (234 m^2^/g). The same trend is observed for the others parameters (micropore and external areas and total and micropore volumes). In fact, the sample pillared by method 11 presented results slightly higher than the PILC obtained by traditional pillaring method, proving that the former method is highly efficient for the synthesis of pillared clays.

Thus, the amount of pillared clay was increased when the methods 3 (6 days pillaring agent) and 11 (1 day pillaring agent) were followed, using the relation of 1 g of clay to 100 mL of water in the clay suspension (higher dilution). This relation was used because, according to the results of series 1, as larger amounts of water are used in the clay suspension, the surface areas and basal spacings become higher.

### 3.3. Series 3: Increase in the Amount of Pillared Clay

**Figure 4:**
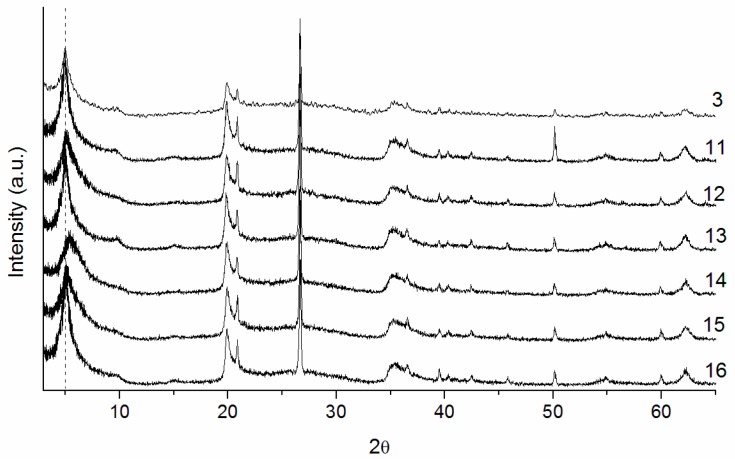
X-ray diffractograms of the series 3 samples.

Figure 4 presents the X-ray diffraction patterns of series 3. Pillarization occurred for all methods except method 14, in which a small shift of the (001) reflection to higher angles of the 2θ axis occurred, indicating a smaller basal spacing in relation to the other samples (Table 8).

For all methods, elevated basal spacings (above 17.5 Å) were obtained, except for method 14, where a value of 16.6 Å was calculated. This small value is due to the concentration used to prepare the pillaring agent (1.5 mol/L) because even after stirring for 13 days, the pillaring solution was still turbid, indicating the formation of agglomerates and other species beyond the Keggin ion [19].

**Figure 5:**
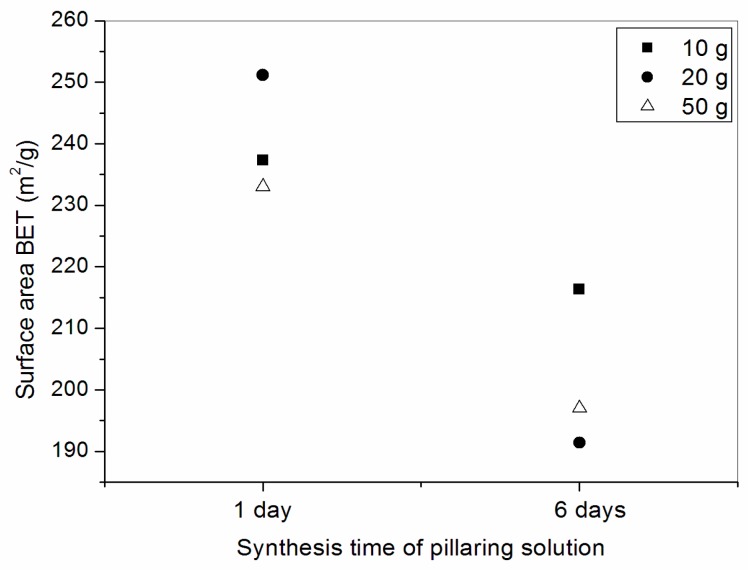
Values of surface area BET to several amounts of pillared clays as a function of time of preparation of the pillaring agent.

From the analysis of the data in Table 8, it could be noticed that when increasing the amount of pillared clay (methods 12, 14 and 15), a slight reduction of surface area occurred, with the lowest surface area obtained by method 14 (179 m^2^/g). For methods 11, 13, and 16, where the pillaring agent was synthesized in the course of a day with a heating stage at 60 °C, the surface areas remained high, even when pillaring 50 g of clay (Figure 5).

All pillared clays synthesized by the pillaring agent prepared in the course of 1 day (methods 11, 13 and 16) obtained surface areas larger than 200 m^2^/g. The clays pillared by the pillaring agent prepared over the course of 6 days obtained lower surface areas. Table 9 presents the data referring to the textural parameters from samples of this series. Not only was the surface area higher for samples pillared by the method using heating, other data such as total pore volume and micropore volume were larger for the samples pillared by this method.

Comparing sample 15 (50 g of clay pillared by the traditional method) with sample 16 (50 g pillared by the method using heating), these two samples show the same basal spacing. However, a higher surface area was obtained for sample 16 (233 m^2^/g); for sample 15, the surface area was 197 m^2^/g. This lower value was most likely due to the pillaring agent utilized, where the concentration of the solution used was 1.2 mol/L, and even after 6 days of aging, the pillaring solution showed a little turbidity. Because method 16 used heating during the synthesis of the pillaring agent, no turbidity occurred, and the solution was completely clear when used, not presenting the problems reported in methods 14 and 15.

Therefore, the method of synthesis of the pillaring agent that applies heating at 60 °C with subsequent aging of the pillaring solution for 24 h at ambient temperature is the most suitable for pillaring large quantities of clay, which requires concentrated solutions. In method 16, fifty grams of pillared clay were produced by using this methodology. The basal spacing and surface area obtained (17.6 Å and 233 m^2^/g) are in line with literature results achieved in pillaring procedures employing few grams of clay [14,36]. The traditional method is efficient just for small quantities of clay because increasing the concentration of the pillaring agent creates turbidity, generating other Al species.

## 4. Conclusions

Three series of experiments (water effect, method effect and increase in the amount of pillared clay) were studied in order to verify their influence in the final properties (basal spacing and surface area) of pillared clays. In series 1, diluted clay suspensions (minimum 1 g/50 mL) are required to obtain pillared materials with basal spacings and surface areas superior than 17 Å and 200 m^2^/g, respectively.

In series 2, the most outstanding pillaring methodology employed was method 11, in which the pillaring agent was synthesized in 24 h with heating at 60 °C. The resulting Al-PILC presented basal spacing of 17.5 Å and surface area of 237 m^2^/g.

In series 3, fifty grams of pillared clay with high basal spacing and surface area (17.6 Å and 233 m^2^/g, respectively) were obtained by applying the best pillaring method studied in series 2 (pillaring agent synthesized at 60 °C with subsequent aging over 24 h at ambient temperature) and amount of water in clay suspension (1 g of clay/100 mL of water). The textural properties and basal spacing obtained by this material are comparable to the results obtained with the traditional pillaring method employing just 3 g of clay.

## Figures and Tables

**Table 1:** Modified parameters to evaluate the effect of water.

Method	Solution Concentrations	Relation of Clay/Water	Amount of Pillared Clay
Traditional	0.2 mol/L	1 g/100 mL	3 g
1	0.6 mol/L	1 g/10 mL	10 g
2	0.6 mol/L	1 g/50 mL	10 g
3	0.6 mol/L	1 g/100 mL	10 g
4	0.6 mol/L	1 g/100 mL	10 g
5	0.6 mol/L	1 g/100 mL	10 g

**Table 2:** Modified parameters to study the effect of the method.

Method	Solution Concentrations	Relation of Clay/Water	Amount of Pillared Clay
Traditional	0.2 mol/L	1 g/100 mL	3 g
6	0.6 mol/L	-	3 g
7	0.6 mol/L	1 g/100 mL	10 g
8	0.6 mol/L	-	10 g
9	0.2 mol/L	1 g/100 mL	3 g
10	0.2 mol/L	1 g/50 mL	3 g
11	0.6 mol/L	1 g/100 mL	10 g

**Table 3:** Modified parameters to increase the amount of pillared clay.

Method	Solution Concentrations	Amount of Pillared Clay	Observation
Traditional	0.2 mol/L	3 g	Pil. Ag. 6 days ^a^
3	0.6 mol/L	10 g	Pil. Ag. 6 days
11	0.6 mol/L	10 g	Pil. Ag. 60 °C, 24 h ^b^
12	1.2 mol/L	20 g	Pil. Ag. 6 days
13	1.2 mol/L	20 g	Pil. Ag. 60 °C, 24 h
14	1.5 mol/L	50 g	Pil. Ag. 6 days
15	1.2 mol/L	50 g	Pil. Ag. 6 days
16	1.2 mol/L	50 g	Pil. Ag. 60 °C, 24 h

^a^ Pillaring agent prepared over 6 days; ^b^ pillaring agent prepared at 60 °C in 24 h.

**Table 4:** Modified parameters, basal spacings and surface area (BET) values of series 1 samples.

Method	Relation of Clay/Water (g/mL)	Mass of Clay	Amount of Water in Clay Suspension	d_001_ (Å)	S_BET_ (m^2^/g)	Observations
Natural	-	-	-	15.1	58	-
Traditional	1/100	3 g	300 mL	17.8	234	-
1	1/10	10 g	100 mL	16.8	142	-
2	1/50	10 g	500 mL	17.5	217	-
3	1/100	10 g	1000 mL	17.9	216	-
4	1/100	10 g	1000 mL	17.8	150	−500 mL
5	1/100	10 g	1000 mL	17.3	149	−900 mL

**Table 5:** Textural parameters evaluated by N_2_ physisorption isotherms of series 1 samples.

Method	S_BET_ (m^2^/g)	S_micro_ (m^2^g)	S_ext_ (m^2^/g)	V_total_ (cm^3^/g)	V_micro_ (cm^3^/g)
Natural	58	19	39	0.070	0.010
Traditional	234	195	39	0.146	0.100
1	142	115	27	0.093	0.059
2	217	180	37	0.135	0.093
3	216	180	36	0.131	0.092
4	150	125	25	0.098	0.064
5	149	120	29	0.101	0.062

**Table 6:** Modified parameters, basal spacings and surface area values of series 2 methods.

Method	Relation of Clay/Water (g/mL)	Mass of Clay	Amount of Water in Clay Suspension	d_001_ (Å)	S_BET_ (m^2^/g)	Observations
Natural	-	-	-	15.1	58	-
Traditional	1/100	3 g	300 mL	17.8	234	-
6	-	3 g	-	13.7	85	Refluxed
7	1/100	10 g	1000 mL	17.2	201	1 month
8	-	10 g	-	17.1	71	In Situ
9	1/100	3 g	300 mL	12.9	74	In Situ w/exp ^a^
10	1/50	3 g	150 mL	17.7	225	Pil. Ag. 1 day ^b^
11	1/100	10 g	1000 mL	17.5	237	Pil. Ag. 1 day

^a^ In situ with expansion; ^b^ pillaring agent prepared over 1 day.

**Table 7:** Textural parameters evaluated by N_2_ physisorption isotherms of series 2 samples.

Method	S_BET_ (m^2^/g)	S_micro_ (m^2^/g)	S_ext_ (m^2^/g)	V_total_ (cm^3^/g)	V_micro_ (cm^3^/g)
Natural	58	19	39	0.070	0.010
Traditional	234	195	39	0.146	0.100
7	201	169	32	0.122	0.087
10	225	184	41	0.140	0.095
11	237	196	41	0.147	0.102

**Table 8:** Modified parameters, basal spacings and surface areas of series 3 samples.

Method	Solution Concentrations	Relation Clay/Water (g/mL)	Mass of Clay	d_001_ (Å)	S_BET_ (m^2^/g)	Observation (Pillaring Agent)
Natural	-	-	-	15.1	58	-
Traditional	0.2 mol/L	1/100	3 g	17.8	234	6 days
3	0.6 mol/L	1/100	10 g	17.9	216	6 days
11	0.6 mol/L	1/100	10 g	17.6	237	60 °C, 24 h
12	1.2 mol/L	1/100	20 g	17.7	191	6 days
13	1.2 mol/L	1/100	20 g	17.5	251	60 °C, 24 h
14	1.5 mol/L	1/100	50 g	16.6	179	6 days
15	1.2 mol/L	1/100	50 g	17.6	197	6 days
16	1.2 mol/L	1/100	50 g	17.6	233	60 °C, 24 h

**Table 9:** Textural parameters of series 3 samples.

Sample	S_BET_ (m^2^/g)	S_micro_ (m^2^/g)	S_ext_ (m^2^/g)	V_total_ (cm^3^/g)	V_micro_ (cm^3^/g)
Natural	58	19	39	0.070	0.010
Traditional	234	195	39	0.146	0.100
3	216	180	36	0.131	0.092
11	237	196	41	0.147	0.102
12	191	154	37	0.122	0.079
13	251	207	44	0.156	0.106
14	179	137	42	0.122	0.071
15	197	157	40	0.127	0.080
16	233	199	34	0.143	0.102
